# Strategies to target the cancer driver MYC in tumor cells

**DOI:** 10.3389/fonc.2023.1142111

**Published:** 2023-03-08

**Authors:** Leonie I. Weber, Markus Hartl

**Affiliations:** Institute of Biochemistry and Center of Molecular Biosciences (CMBI), University of Innsbruck, Innsbruck, Austria

**Keywords:** transcriptional regulation, protein-protein interaction, cell proliferation, tumorigenesis, cell penetrating peptides, nanoparticles

## Abstract

The MYC oncoprotein functions as a master regulator of cellular transcription and executes non-transcriptional tasks relevant to DNA replication and cell cycle regulation, thereby interacting with multiple proteins. MYC is required for fundamental cellular processes triggering proliferation, growth, differentiation, or apoptosis and also represents a major cancer driver being aberrantly activated in most human tumors. Due to its non-enzymatic biochemical functions and largely unstructured surface, MYC has remained difficult for specific inhibitor compounds to directly address, and consequently, alternative approaches leading to indirect MYC inhibition have evolved. Nowadays, multiple organic compounds, nucleic acids, or peptides specifically interfering with MYC activities are in preclinical or early-stage clinical studies, but none of them have been approved so far for the pharmacological treatment of cancer patients. In addition, specific and efficient delivery technologies to deliver MYC-inhibiting agents into MYC-dependent tumor cells are just beginning to emerge. In this review, an overview of direct and indirect MYC-inhibiting agents and their modes of MYC inhibition is given. Furthermore, we summarize current possibilities to deliver appropriate drugs into cancer cells containing derailed MYC using viral vectors or appropriate nanoparticles. Finding the right formulation to target MYC-dependent cancers and to achieve a high intracellular concentration of compounds blocking or attenuating oncogenic MYC activities could be as important as the development of novel MYC-inhibiting principles.

## 1 MYC: An oncogenic transcription factor with pleiotropic functions

### 1.1 The *MYC* oncogene and its protein product

**Figure 1:**
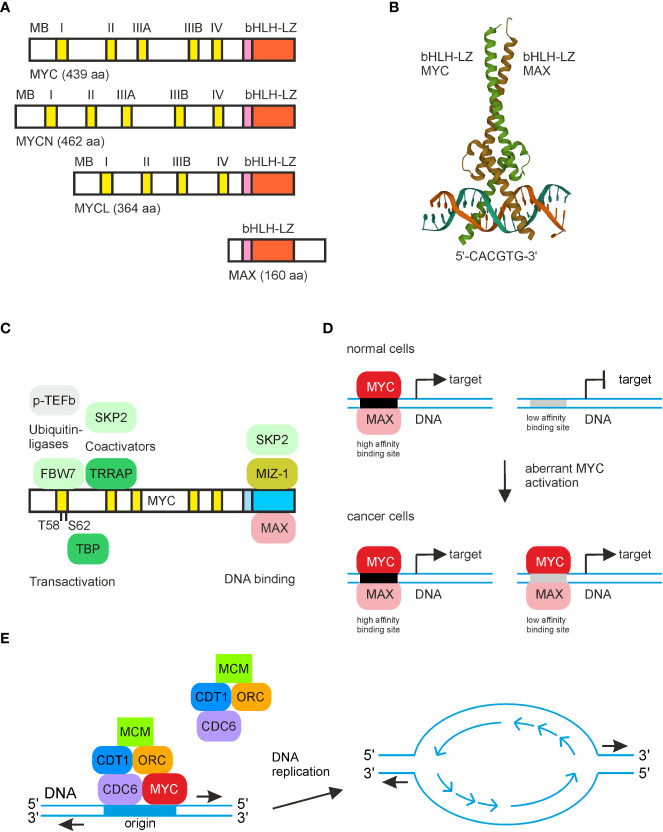
MYC structure and relevant protein and DNA interactions. **(A)** Schematic depiction of the human MYC paralogs MYC (c-Myc), MYCN (N-Myc), and LMYC (L-Myc) and of human MAX. The conserved dimerization and DNA binding regions (bHLH-LZ) in the carboxyl-terminal regions are depicted in pink/orange, and the MYC boxes (MB) in the amino-terminal transactivation regions are in yellow. **(B)** Structure of the human MYC/MAX bHLH-LZ domains binding to the E-box (5′-CACGTG-3′). The image was created from the PDB entry 1NKP. **(C)** Selected MYC protein interaction partners and their positions where they bind the MYC protein surface. **(D)** Simplified illustration of the transcriptional amplification model. At low MYC concentrations, only high-affinity binding sites are occupied, leading to transcriptional regulation of target genes. At high MYC levels, as it occurs in most cancer cells, also targets with low-affinity binding sites are bound by MYC, causing aberrant expression of cell transformation-associated MYC target genes. **(E)** Scheme showing MYC interactions with proteins of the pre-replicative complex at the onset of DNA replication leading to initiation of bidirectional DNA synthesis.

*MYC* gene is one of the most frequently deregulated oncogenes in many cancer types and a hallmark in the majority of human cancers (1). The v-*myc* oncogene was originally discovered as the transforming principle in avian leukemogenic retroviruses (2–5). The gene regulator MYC (c-MYC) and its paralogs MYCN (N-Myc) and MYCL (L-Myc) are bHLH-LZ proteins encompassing a protein dimerization domain (helix-loop-helix, leucine zipper) and a DNA contact surface (basic region), which are both located in the carboxyl-terminus (**Figure 1**). The amino-termini of MYC proteins mediate crucial cellular functions including transcriptional regulation. MYC proteins form heterodimers with the MYC-associated factor X (MAX) protein typically binding to a canonical DNA sequence element termed E-box in the promoter regions of multiple MYC target genes (6) (**Figure 1**). The bHLH-LZ proteins MYC and MAX are evolutionary conserved having functional homologs in primitive metazoans and pre-metazoans (6–8), suggesting that basic functions of MYC arose very early in the evolution of multicellular organisms.

MYC represents the hub of a network controlling the expression of approximately 30% of the human genes and regulates fundamental cellular processes like growth, proliferation, differentiation, metabolism, and apoptosis (6). Acting mainly as a transcription factor, the principal function of MYC is specific gene regulation, but MYC also executes additional tasks implicated in DNA replication and chromatin remodeling (6, 9). Therefore, MYC facilitates the assembly of protein complexes at the origin of replication (ORI) by interacting with proteins from the pre-replicative complex like CDT1, whose underlying gene itself is a transcriptional MYC target (10–12) (**Figure 1**). Overexpression of MYC enhances replication origin activity, which subsequently induces replicative stress leading to DNA damage and checkpoint activation pointing to a critical MYC function in DNA synthesis, which is aberrantly activated in cancer cells (10, 13).

Due to the regulation of thousands of genes, MYC can be regarded as a globally acting transcription factor (14) binding with its C-terminal bHLH-LZ region sequence specifically to double-stranded DNA in the relevant target promoter regions. The N-terminal transactivation domain of MYC contains several conserved motifs termed MYC boxes, from which MYC box II binds to chromatin-remodeling co-activators being components of several histone acetyltransferase (HAT) complexes (15) (**Figure 1**). MYC also interacts with the TATA box binding protein (TBP) and with other basal transcription factors leading to re-activation of paused RNA polymerase II upon phosphorylation, indicating that MYC has specific and general functions in transcriptional activation (14, 15). So far, it has not been possible to ascribe the oncogenic properties of MYC to a defined and complete set of target genes, and results from recent investigations suggest that the principal MYC function exceeds that of a typical sequence-specific binding gene regulator (14, 15). Instead, MYC may act as a universal amplifier of gene expression rather than regulating a distinct set of target genes. Accordingly, in tumor cells, the promoters of all actively transcribed genes are activated by MYC leading to non-linear amplification of pre-existing transcriptional activities (14) (**Figure 1**). The amplifier model also explains how ectopic MYC increases the efficiencies of other transcription factor programs, suggesting that MYC acts as a superior master switch in global transcriptional regulation (11). Furthermore, recent comprehensive analyses of gene expression revealed that during MYC-initiated tumorigenesis, gene expression changes follow a distinct pattern, which is associated with embryonic, ribosomal biogenesis, and tissue-lineage dedifferentiation processes (16).

Concerning MYC-repressed genes, which are almost as numerous as the upregulated ones, there exist additional MYC-associated repressive mechanisms. MYC interacts with other transcription factors such as MIZ-1 or SP1 converting MYC from a transcriptional activator into a transcriptional repressor (6, 14, 15, 17) (**Figure 1**). Many MYC-repressed genes are involved in cell cycle regulation where MYC downregulates the expression of distinct cell cycle inhibitors or genes mediating cell growth arrest (18). Interactions of MYC with MIZ-1 or SP1, and chromatin accessibility influenced by epigenetic modifications could lead to specific target gene repression, thereby defining a more selective amplification model (19). Alternatively, gene repression may be more indirect and caused by MYC-induced upregulation of repressive components and other repressive mediators like distinct microRNAs (14, 15). Transcriptional transactivation of MYC/MAX complexes is further antagonized by MAX/MXD1 heterodimers that compete for E-box binding. These protein complexes repress canonical targets by recruitment of histone deacetylases (HDACs) and corepressors leading to chromatin closing (6, 15).

Multiple proteins have been identified, which interact with MYC and enable MYC for processes such as promoter DNA binding, chromatin modification, or regulation of gene transcription. In fact, the combination of multiple protein–protein interactions is a prerequisite for MYC in order to function as an oncoprotein. MYC interactions occur through conserved regions such as the transactivation domain containing the MYC homology boxes (MB), the basic region, and the helix-loop-helix-leucine-zipper region (bHLH-LZ) (18) (**Figure 1**). On the way to achieving a comprehensive view including the dynamics of components regulating MYC-dependent transcription and DNA replication in a quantitative manner, a model has been recently proposed concerning the differential partitioning and trafficking of the largely unstructured MYC protein. In this model, MYC is part of an interaction network between multiple gene–regulatory complexes and factors, thereby energetically modulating the transcriptional process (11).

### 1.2 MYC-associated signaling pathways

MYC is frequently found deregulated in cancer cells, which are, apart from tumorigenesis-caused genomic instability and epigenetic reprogramming, featured by aberrant activation of proto-oncogenes or inactivation of tumor suppressor genes. In normal tissues, growth-promoting signals are carefully controlled leading to cellular homeostasis, whereas in cancer cells, these functions are derailed. Growth-promoting signals are largely transmitted by growth factors, which bind to cell surface receptors containing intracellular tyrosine kinase domains. From here, the signal branches into multiple and complex transduction pathways mediated mainly by serine/threonine protein kinases to regulate cell cycle progression, cell growth, survival, and energy metabolism. This is then finally accomplished by transcription factors representing the nuclear endpoints of cellular signaling (20). Hence, all key players in cellular signal transduction are encoded by genes, which are normally required to coordinate proper cell metabolism, proliferation, and differentiation, and in many cases, their relevant functions had been elucidated after their identification as transforming principles in highly oncogenic avian and murine retroviruses carrying mutated versions in their genomes (2, 3, 5).

**Figure 2:**
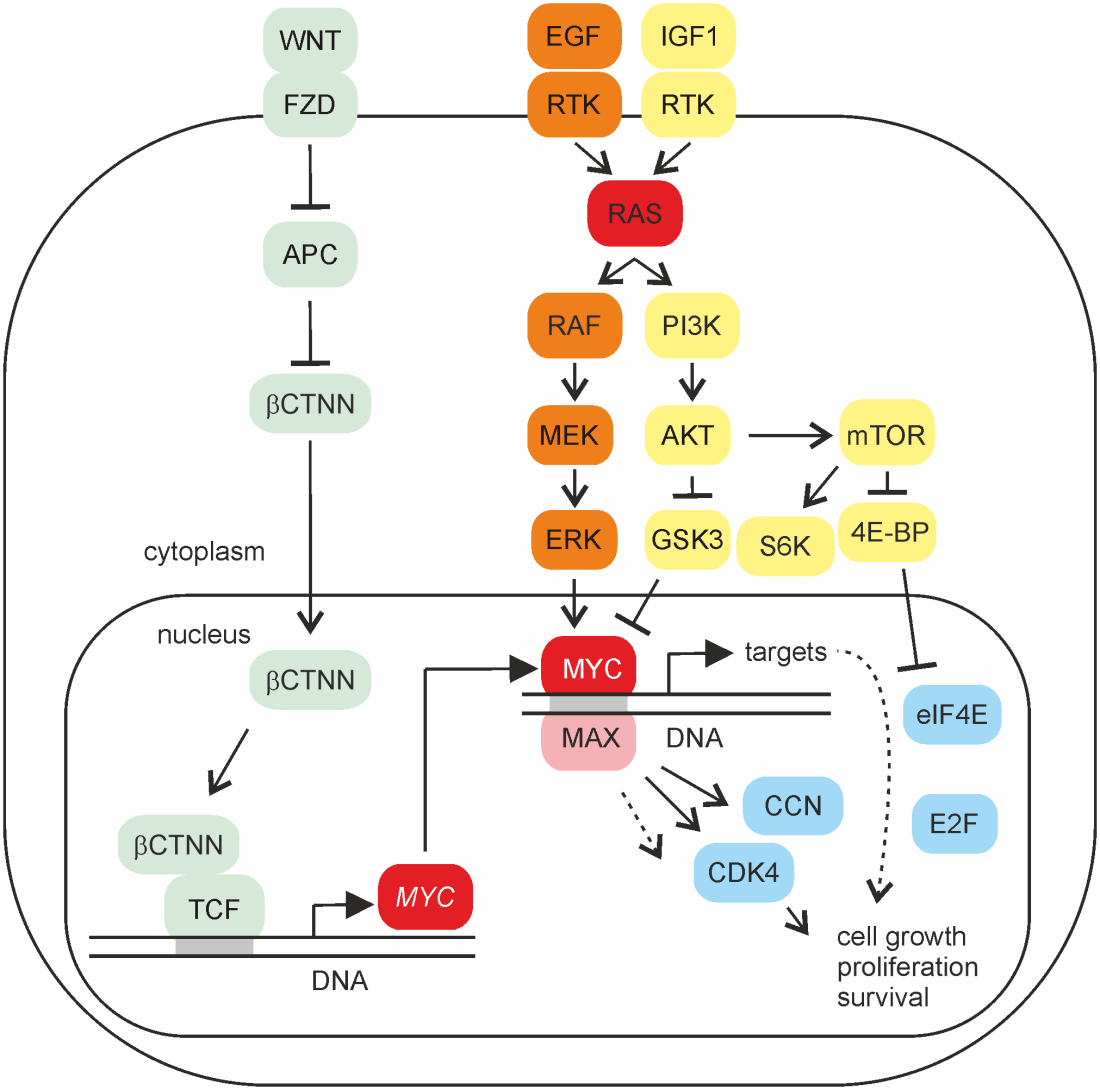
MYC signaling. Schematic depiction of selected signaling pathways activating *MYC* gene and its protein product. Proteins of the WNT, MAPK, or PI3K pathways are shaded in light green, orange, or yellow, respectively. Whereas WNT/β-catenin signaling leads to transcriptional activation of *MYC* gene, MAPK or PI3K signaling has an impact on the post-translational modification of the MYC protein, thereby also influencing its stability. The MYC/MAX heterodimer binds to DNA and regulates the expression of multiple specific target genes. Selected protein products of distinct MYC targets regulating the cell cycle are shaded in blue.

Representing an effector of multiple signaling pathways, MYC acts as a master switch in cell proliferation and differentiation. In particular, the mitogen-activated protein kinase (MAPK) and the phosphatidylinositol-3-phosphate kinase (PI3K) pathways mediating cell growth and survival end in activation of the nuclear MYC protein at the post-translational level (21–23) (**Figure 2**). Both signaling routes synergistically regulate MYC protein stability by phosphorylation of distinct residues in MYC box I (MBI), one of several conserved MYC segments in the transactivation domain, leading to MYC accumulation during the initial stage of cell proliferation (24, 25). The MBI in the transactivation domain of MYC contains a canonical phosphodegron with two interdependent phosphorylation sites critical for the regulation of MYC stability and function. From the remaining MYC boxes, MBII contributes to gene activation and protein degradation, whereas MBIII and MBIV are implicated in transcriptional repression and apoptosis (26). Multiple transcriptional target genes of MYC subsequently promote cell division and proliferation.

In MAPK signaling, the serine/threonine protein kinase RAF and its paralogues ARAF and BRAF are taking a key position resulting in the phosphorylation of master gene regulators such as MYC or the transcription factor AP-1 (2, 27–29) (**Figure 2**). Further downstream of RAF, the MAP kinase ERK phosphorylates the serine residue 62 in MBI leading to MYC protein stabilization. However, phosphorylation of threonine 58 in MBI mediated by glycogen synthase kinase 3 (GSK3) is associated with MYC degradation (24). Consequently, inhibition of GSK3 by protein kinase AKT-mediated phosphorylation within the PI3K pathway leads to the stabilization of the MYC protein. In addition to GSK3, AKT phosphorylates distinct FOXO transcription factors, which normally impede the induction of critical MYC target genes required for cell proliferation (30–32). The third downstream substrate inhibited by AKT is a component of the tuberous sclerosis complex (TSC) termed TSC2. Phosphorylation of TSC2 relieves TSC-mediated inhibition of the RHEB protein and leads to activation of the mammalian target of rapamycin (mTOR) component mTORC1. Canonical targets of mTORC1 are the ribosomal protein S6 kinase (S6K) and the eukaryotic translation initiation factor 4E-binding protein (4E-BP1) (**Figure 2**). 4E-BP1 is a master regulator of protein synthesis control, required for cancer cell survival in MYC-dependent tumorigenesis (33). Phosphorylation of S6K and 4E-BP1 stimulates mRNA translation of distinct transcription factors including MYC (31), thereby increasing the levels of this oncoprotein. Possible links between MYC and mTOR are provided by recent results showing that phosphorylation of AKT by the mTOR component mTORC2 directly regulates MYC expression (34). In addition, mTOR and AKT stimulate MYC expression by post-transcriptional modification of distinct substrate proteins or *via* activation of MYC target genes to support translation for cell growth and proliferation (21).

### 1.3 The role of MYC in human cancer

In normal cells or tissues, MYC is tightly regulated at transcriptional, translational, and post-translational levels leading to a half-time of approximately 20 min (35, 36). However, in many cancers like lymphomas or carcinomas, *MYC* gene is aberrantly activated by transcriptional deregulation, gene amplification, chromosomal translocation, or post-translational modification (6, 37). In fact, the deregulation of *MYC* gene and concomitantly of multiple MYC targets is a frequent event in tumorigenesis occurring in approximately 70% of all human cancer cell types, indicating that aberrant MYC expression drives the genesis of many tumors (4, 6). This results in the deregulation of cell cycle progression, metabolism, differentiation, and angiogenesis, which then contributes to neoplastic transformation. Since many tumors depend on continuous MYC expression, this oncoprotein is one of the crucial drivers in human cancers, from which many are associated with a poor clinical outcome (15), indicating that deregulated MYC contributes to or drives the genesis of multiple tumors (4, 6, 38).

Transcriptional deregulation of the human *MYC* gene was first observed in Burkitt’s lymphoma, where the *MYC* proto-oncogene (c-*myc*) is translocated into the immunoglobulin heavy chain locus (3, 6). Transgenic mice overexpressing *MYC* alleles in lymphoid or myeloid cells develop lymphoma or leukemia (39). MYC upregulation in lymphoma is frequently accompanied by additional translocations leading to the inactivation of the BCL6 repressor and activation of the BCL2 oncoprotein (40). In T-cell lymphoma, the MYC protein is overexpressed without *MYC* gene rearrangement or amplification but stabilized by Ca^2+^/calmodulin-dependent protein kinase II γ (CAMKIIγ) phosphorylation (41). In chronic myeloid leukemia (CML), the tumor suppressor TP53 (p53) and the cancer driver MYC mediate the CML network triggered by the fusion protein BCR-ABL, which displays constitutive tyrosine kinase activity. Perturbation of this network using compounds stabilizing TP53 and inhibiting MYC transcription results in synergistic cell killing and differentiation, thereby preventing CML formation (42). In acute lymphoblastic and myeloid leukemia (ALL and AML), MYC is often overexpressed and frequently associated with disease progression (39). Hence, MYC represents a transcription factor with a pivotal role in hematopoiesis and blood cancer. However, MYC is also implicated in multiple other tumor forms. In breast, ovarian, and endometrial cancers, *MYC* amplification is a characteristic feature with poor prognosis (1). Amplification of *MYC* gene also occurs in colon cancer, apart from direct transcriptional *MYC* activation caused by the WNT/β-catenin signaling pathway (**Figure 2**) (1, 43). Furthermore, inactivating mutations of the E3 ubiquitin ligase FBXW7 lead to increased MYC protein stability (1, 44). Likewise, in lung and pancreatic cancers, *MYC* and *MYCN* genes are frequently amplified (1).

Deregulated MYC is also implicated in breast and prostate cancers, which belong to the most frequent malignancies in women and men, respectively, with one-eighth of the entire human population developing one of these highly heterogeneous cancer forms. In these tumors, *MYC* is deregulated due to aberrant transcriptional activation, gene amplification, or mRNA/protein stabilization correlated with a loss of BRCA or TP53 tumor suppressors, which normally inhibit MYC’s transcriptional and transforming activities. Breast cancer can be divided into three clinical subtypes, featured by expression of the estrogen receptor (ER), the progesterone receptor (PR), or amplification of the *HER-2/NEU* gene encoding an Erb-type receptor tyrosine kinase, which can be all treated with chemotherapy or specific tyrosine kinase inhibitors. Tumors that do not express one of these markers are classified as triple-negative breast cancer (TNBC). TNBC is characterized by a basal-like tumor subtype with no functional BRCA1 or TP53 and a lack of appropriate therapeutic targets, which could be reached by clinically approved inhibitors (45–47). These tumors are furthermore featured by overexpression of MYC proteins, elevated MYC-driven pathways, and deregulated MYC-dependent gene signatures (47–49). In addition, MAPKs inducing MYC oncogenicity are frequently upregulated in chemotherapy-resistant cells and involved in the resistance toward the ER antagonist tamoxifen (50).

In advanced stages of prostate cancer, hormonal therapies using steroidal compounds like enzalutamide are applied with the aim to block androgen production and to reduce the activities of androgen receptor signaling (51). However, despite initial success, tumors often recur and even develop into castration-resistant prostate cancer (CRPC) with poor prognosis (51, 52). During progression, the androgen receptor becomes aberrantly upregulated along with the hyperactivation of distinct oncogenes, including amplification and overexpression of *MYC* (53). Therefore, MYC antagonizes and deregulates the transcriptional androgen receptor program (53) and could represent the driving force in the transition toward therapy resistance. In metastatic prostate tumor progression, *MYC* gene amplification and upregulated MYC expression caused by the activation of upstream-acting kinase pathways are considered the main events (54). MYC inhibition sensitizes enzalutamide-resistant cells toward growth inhibition by this drug, suggesting that MYC targeting may be useful in androgen receptor-directed therapy (55). In advanced prostate adenocarcinoma, MYC signaling is one of the most activated pathways (56), and alterations in *MYC* gene loci occur already in the early phase. In metastatic prostate tumor progression, *MYC* gene amplification or upregulation of *MYC* expression caused by activation of upstream-acting kinase pathways are considered the main events (54, 57).

### 1.4 Current strategies to inhibit oncogenic MYC

**Figure 3:**
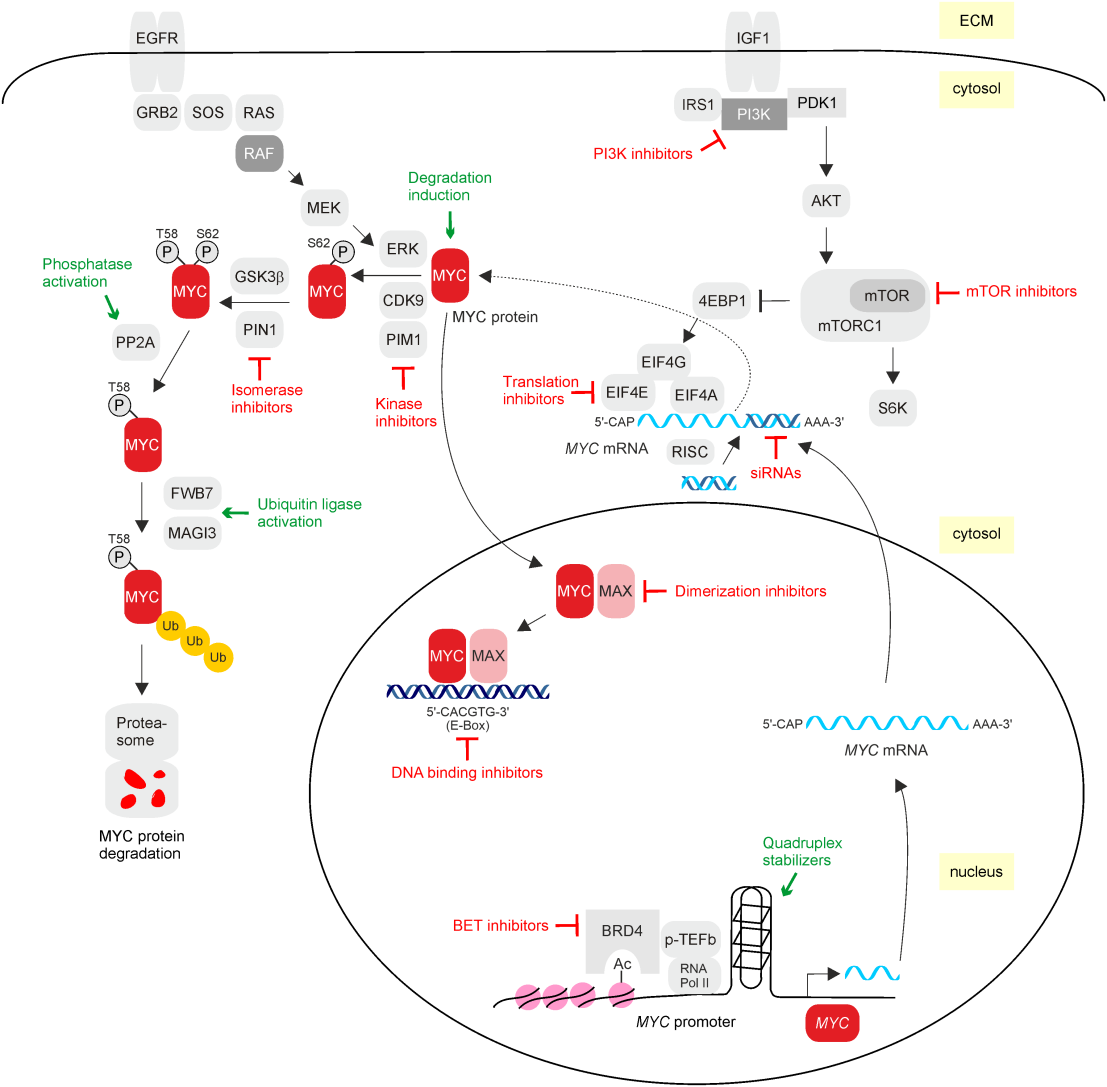
MYC inhibitors. Overview of direct and indirect possibilities to interfere with the activity of oncogenic MYC. MYC can be inhibited on the transcriptional level by preventing *MYC* mRNA expression, post-transcriptionally by preventing mRNA translation, post-translationally by interfering with upstream signaling or by inducing premature protein degradation, and finally by blocking dimerization with MAX and/or DNA binding, thereby impairing oncogenic target gene expression. Inhibitory effects on MYC are depicted in red, and events that activate MYC-inhibitory pathways are in green. Relevant molecules interfering with oncogenic MYC activity are listed in **Table 1**.

MYC is one of the most commonly deregulated proteins in multiple cancer cells but is difficult to target with appropriate drugs. As outlined above, aberrant MYC activity is observed in the majority of human tumors caused by transcriptional deregulation, gene amplification, or chromosomal translocation of *MYC* gene or by post-translational modifications of the MYC protein (6). Due to MYC’s pivotal role in human cancer, it is not surprising that many attempts have been pursued to inhibit oncogenic *MYC* functions (58) including multiple approaches to interfere with its transcriptional or post-translational regulation (25, 35) (**Figure 3**). However, vertebrate MYC has two paralogues (MYCN and MYCL) with partially redundant functions, which need to be targeted as well in order to achieve an efficient therapeutic effect. A further complication is that MYC has an important role in distinct physiological processes linked to tissue regeneration, suggesting that complete MYC inhibition could also affect normal homeostasis (26), although it has been shown that efficient MYC inhibition is tolerated surprisingly well (59). In the following sections, different types of MYC-inhibiting molecules are discussed. Despite all difficulties in effectively inhibiting MYC in tumor cells, genetic models indicate that MYC inhibition could be supportable for an organism and lead to sustainable tumor regression (35).

To inhibit MYC’s oncogenic activities in cancer cells, different types of molecules have been applied like organic molecules, nucleic acids, peptides, or natural compounds derived from plants (**Table 1**). However, MYC is a transcription factor majorly present in the nucleus, and in contrast to oncogenic enzymes, which can be tightly bound by inhibiting molecules, MYC is an intrinsically unstructured non-enzymatic protein with no particular surface, supporting efficient docking of an inhibiting molecule (107). Consequently, MYC is hardly accessible by conventional small molecules or specific antibodies, which is the reason why numerous clinically approved drugs against oncogenic kinases exist but not against oncogenic transcription factors such as MYC.

**Table 1:** Molecules interfering with oncogenic MYC activity.

Mode of action	Name	Mechanism	Clinical status	Reference
**MYC/MAX dimerization**	KJ-Pyr-9	Prevents MYC dimerization	Preclinical, tested *in vivo*	(60)
	KSI-3716	Prevents promoter binding of the MYC/MAX dimer	Preclinical, tested *in vivo*	(61)
	L755507	Prevents MYC/MAX dimerization	Preclinical, tested *in vitro*	(62)
	ME-47	Disrupts MAX/E-box binding	Preclinical, tested *in vivo*	(63)
	MI3-PD	Prevents MYC/MAX dimerization	Preclinical, tested *in vivo*	(64)
	MYCi 975	Prevents MYC/MAX dimerization, promotes T58 phosphorylation	Preclinical, tested *in vivo*	(65, 66)
	MYCMI-6	Prevents MYC/MAX dimerization	Preclinical, tested *in vivo*	(67)
	Mycro3	Prevents MYC/MAX dimerization	Preclinical, tested *in vivo*	(68)
	Omomyc	Prevents MYC/MAX dimerization	Clinical trial phase I	(69–71)
	RASSF7	Competes with MAX, MYC destabilization	Preclinical, tested *in vitro*	(72)
***MYC* transcription inhibition**	dBET1	Degrades BRD4	Preclinical, tested *in vivo*	(73, 74)
	IZCZ-3	Stabilizes MYC G-quadruplex	Preclinical, tested *in vivo*	(75)
	JQ1	BRD4 inhibitor	Preclinical, tested *in vivo*	(76–78)
	Morpholino-thienopyrane	BRD4 and PI3K inhibitor	Preclinical, tested *in vivo*	(79)
	QN-1	Stabilizes MYC G-quadruplex	Preclinical, tested *in vivo*	(80)
	SF1126	BRD4 and PI3K inhibitor	Preclinical, tested *in vivo*	(81)
	Thiazole peptide TH3	Stabilizes MYC G-quadruplex	Preclinical, tested *in vitro*	(82)
	THZ1	CDK7 inhibitor	Preclinical, tested *in vivo*	(83, 84)
**MYC translation inhibition**	CUDC-907	PI3K and HDAC inhibitor	Preclinical, tested *in vivo*	(85)
	Omacetaxine	Translation inhibitor	FDA-approved	(86)
	Rapamycin	mTOR inhibitor	Preclinical, tested *in vivo*	(87)
	Silvestrol	eIF4E inhibitor	Preclinical, tested *in vivo*	(88)
**MYC protein stability**	ATRA	PIN1 inhibitor	Clinical trial phase II	(89)
	AZD1208	PIM kinase inhibitor	Clinical trial phase I	(90, 91)
	DT1154	PP2A activator	Preclinical, tested *in vivo*	(92)
	Curcumin	MYC cross-linking	Preclinical, tested *in vitro*	(93)
	Fludarabine phosphate	MYC destabilization	Preclinical, tested *in vivo*	(94)
	KPT-6566	PIN1 inhibitor	Preclinical, tested *in vivo*	(95)
	MAGI3 overexpression	Ubiquitin ligase	Preclinical, tested *in vivo*	(96)
	MLN8237	Aurora-A kinase inhibitor	Clinical trial phase III	(97, 98)
	Momordin Ic	SENP1 inhibitor	Preclinical, tested *in vivo*	(99)
	OP449	PP2A activator	Preclinical, tested *in vivo*	(100)
	Pimi	PIM kinase inhibitor	Preclinical, tested *in vitro*	(101)
	Sulfopin	PIN1 inhibitor	Preclinical, tested *in vivo*	(102)
	TD-19	CIP2A inhibitor	Preclinical, tested *in vivo*	(103)
	TD-52	CIP2A inhibitor	Preclinical, tested *in vivo*	(104)
	TRAFTAC	MYC degradation	Preclinical, tested *in vivo*	(105)
	UNC10112785	CDK9 inhibitor	Preclinical, tested *in vitro*	(106)

The apparently undruggable MYC protein structure promoted the development of alternative inhibiting principles to achieve desirable anti-tumor effects. These encompass disruption of the MYC/MAX complex by small organic molecules (60, 108) or by appropriate peptides like the dominant negative Omomyc (26, 109). Progress has been also made by the development of small-molecule inhibitors inducing epigenetic silencing or disrupting MYC/MAX DNA-binding activities to prevent further activities occurring downstream of MYC. In addition, the application of synthetic lethality effects associated with MYC overexpression has been successfully pursued (110), as well as the targeting of specific protein interactions with one of the multiple MYC binding partners (18, 111). Blocking specific protein kinases regulating MYC at the post-translational level or interfering with the functions of transformation-associated MYC target genes represents further options. Due to the complex regulation of MYC signaling, combinations of different inhibition modes (**Figure 3**) may provide a more effective response (35). Most of the applied strategies for *MYC* gene or MYC protein inhibition are still tested in preclinical or early-phase clinical stages, and only a few have yet succeeded in advanced clinical trials (112) (**Table 1**). Hence, MYC is still classified as a “difficult-to-drug” or even “undruggable” therapeutic target (113). All current possibilities to directly or indirectly inhibit oncogenic MYC are comprehensively summarized in recent excellent reviews (18, 109, 114). A description and illustration of selected principles and methods to inhibit MYC (**Figure 3**) are given below.

#### 1.4.1 Interference with dimerization and DNA binding

In order to prevent oncogenic activities from occurring downstream of MYC, inhibition of MYC/MAX dimerization and subsequent DNA binding is straight-forward since this blockade is independent of upstream alterations in cellular signaling (35). However, the structural heterogeneity of MYC based on its intrinsically disordered structure makes the design of appropriate drugs challenging. Even the MYC/MAX interaction surface is relatively large and flat, making it difficult to identify binding sites for small-molecules (115). Nevertheless, multiple small organic molecules interfering with MYC/MAX dimerization have been developed (108) like pyridine derivatives, leading to inhibition of transcriptional activation and oncogenesis (60, 116) or other types of compounds (62, 67, 116, 117) including stabilizers of MAX homodimers to reduce the availability for MYC (118). Likewise, small molecules disrupting MYC-associated protein–protein and protein–DNA interactions interfering with MYC-dependent transcription and oncogenesis have been developed and characterized (26, 35, 119). However, most of these compounds suffer from non-adequate pharmacokinetic properties and consequently lack potency in *in vivo* systems (65, 120). To obtain compounds with better pharmacokinetic properties, the chemical space in drug screening has been enlarged, leading to the identification of novel molecules with desired properties. A recently developed small molecule inhibiting MYC is the compound MYCMI-6, which was identified in a cell-based protein interaction screen. This compound blocks MYC-driven transcription and MYC-dependent tumor cell growth in a nanomolar range and binds with high affinity to the bHLH-LZ domain of MYC (**Figure 3**). Furthermore, it inhibits MYC/MAX interaction and induces apoptosis in tumor tissue derived from a MYC-driven xenograft tumor model. An advantage is that this compound does not affect *MYC* expression, making it an interesting candidate to specifically target MYC/MAX with potentially few side effects (67). Another novel compound termed MYCi975 represents a promising specific MYC inhibitor in TNBC cells. MYCi975 shows favorable pharmacokinetics and *in vivo* efficacy by disrupting MYC/MAX complexes and promoting threonine 58 phosphorylation followed by MYC degradation (65). Furthermore, critical MYC target gene expression is impaired, and the proliferation of several TNBC cell lines is arrested, in combination with the compound paclitaxel or doxorubicin. This makes MYCi975 an interesting candidate for combined breast cancer chemotherapy (66). With the use of computer-aided drug discovery, further MYC inhibitors have been identified. The compound L755507 efficiently blocks MYC/MAX heterodimerization, leading to decreased MYC target gene expression and induction of programmed cell death in cancer cells (62). A phenoxy-*N*-phenylaniline derivative was shown to interfere with MYC/MAX dimerization, thereby efficiently inhibiting MYC in colorectal cancer cell lines (121).

In addition to small molecules, short polypeptides have been successfully applied to interfere with MYC-specific functions. The small protein ME47 disrupts the MAX:E-box interaction (**Figure 3**), leading to a block of MYC/MAX transcription and inhibition of tumor growth (63). An elegant approach to interfere with MYC/MAX dimerization and subsequent DNA binding has been achieved by expression of the 90-amino acid dominant negative polypeptide Omomyc (69). This peptide encompasses the bHLH-LZ region of MYC with four amino acid substitutions conferring different dimerization properties. That way, Omomyc can form homodimers and heterodimers with MYC or MAX, which has been confirmed by chromatin immunoprecipitation, proximity ligation assay, and double chromatin immunoprecipitation (ReChIP) (70). By competitive inhibition of DNA binding and sequestration of oncogenic MYC, Omomyc inhibits MYC by reducing its ability to bind to specific E-box sequences in the promoters of MYC target genes (69). Overexpression of Omomyc inhibits MYC-mediated transcription and MYC-dependent cell transformation (26) and offers promising therapeutic impact in several cancer mouse models (69). Furthermore, Omomyc has a cell-penetrating capacity and is functional when delivered as a purified polypeptide into cells (69). Delivery efficiency can even be enhanced upon fusion with a penetrating phylomer peptide (FPPa) as shown in triple-negative breast cancer where this fusion peptide effectively inhibits MYC-dependent transcriptional networks, thereby inducing apoptosis. Furthermore, there is a strong synergism between FPPa-Omomyc and chemotherapeutic agents (122). Likewise, the application of the original Omomyc as a cell-penetrating peptide (Omo-103) in patients suffering from solid cancers has provided promising results in a first phase I trial (59).

To overcome possible pharmacological limitations associated with potentially unstructured mini proteins above 60 amino acids in size and to generate a tool box applicable also to other DNA binding proteins, synthetic transcriptional repressors have been designed, which are better structured and shorter in size (123). A synthetic transcriptional repressor derived from the bHLH-LZ domain of MAX cooperatively binds to the consensus E-box with nanomolar affinity as demonstrated by electrophoretic mobility shift assays (EMSAs), thereby competing with MYC/MAX binding. As a consequence, MYC-dependent transcription programs become downregulated at the proteome level, and cell proliferation decreases (123). This non-natural mimetic comprising the minimal DNA-binding helix and the N-terminal portion of the leucine zipper consists of two separate polypeptides, one with the basic helix and the other one with the minimal zipper helix. These peptides are ligated to build a larger tertiary structure in order to form the minimal tetrahelical helix-loop-helix that recognizes an E-box sequence upon dimerization. The advantage of this technology is that a relatively small secondary and tertiary structure-stabilized peptide with a size of only approximately 6 kDa can be obtained (123).

As an alternative to compounds interfering with MYC/MAX dimerization or DNA binding, double-stranded oligonucleotides containing the binding site for a transcription factor represent therapeutic drug candidates to specifically inhibit oncogenic gene regulators such as MYC. However, these unprotected DNA molecules are normally rapidly degraded upon cellular delivery. To ensure the long-lasting effects of these decoy oligonucleotides, structural modifications like intramolecular hairpins or circularization coupled with specific drug delivery methods, such as coated microbubbles or viral vector-mediated gene transfer, are often required (124). For efficient cellular delivery, the fusion with a cell-penetrating peptide (CPP) may also represent an option, as shown previously with the CPP TP10 to attenuate MYC protein levels. In this case, the TP10 peptide was either mixed with the double-stranded decoy oligonucleotide thereby forming a complex through non-covalent electrostatic interactions or added together with a complementary peptide nucleic acid (PNA) sequence to a decoy strand with a nucleotide overhang (125).

#### 1.4.2 Inhibition of MYC transcription

The *MYC* promoter is bound by transcription factors and chromatin components, which are regulated by several upstream signaling pathways. Conventional gene regulators such as SP1 trigger transcription catalyzed by RNA polymerase II followed by binding of so-called FUSE-binding proteins (FBP) to a FUSE element located far upstream of the transcription site and finally by binding of an FBP-interacting repressor (FIR), which then returns transcription to a basal-state level (126). In addition, the Wilms tumor suppressor protein WT1 acts as a context-dependent oncoprotein by binding to the *MYC* promoter as either a transcriptional activator or repressor. This depends on the absence or presence of the transcriptional corepressor brain acid-soluble protein 1 (BASP1) that converts the WT1 oncoprotein into a tumor suppressor, thereby also blocking transcriptional *MYC* activation (127).

In addition to specific transcription factor binding sites, the *MYC* promoter contains G-quadruplex structures representing four-stranded secondary DNA structures characterized by Hoogsteen-bonded guanine tetrads (109). Quadruplex structures are involved in essential genome functions like transcription, replication, genomic stability, or epigenetic regulation (109) and are overrepresented in the promoter regions of proto-oncogenes such as *MYC* (82, 128). The *MYC* promoter contains a 27-mer G-quadruplex within a nuclease hypersensitive element (129) in equilibrium between transcriptionally active double- or single-stranded DNA and a transcriptionally inactive four-stranded form (82). Since the *MYC* promoter is frequently overactivated in many cancer types (6), interference with *MYC* mRNA transcription by stabilizing transcription-inhibitory secondary DNA structures has become an option to silence *MYC* expression (**Figure 3**). For this reason, small-molecule inhibitors have been developed to target deregulated *MYC* transcription by causing epigenetic silencing or by stabilizing non-canonical G-quadruplex structures within the promoter region (35). To overcome the generally limited selectivity of quadruplex ligands, the development of cell-penetrating thiazole peptides specifically targeting G-quadruplex structures in the *MYC* promoter has been recently reported (82). Therefore, a particular G-quadruplex structure is bound by a crescent-shaped thiazole peptide that enters the nucleus and preferentially stabilizes *MYC* quadruplexes over other promoter G-quadruplexes leading to *MYC* transcription inhibition in cancer cells (82). Another selective binder to the G-quadruplex structure in the *MYC* promoter is the compound QN-1 representing a difluoro-substituted quinoxaline. It has been shown that QN-1 downregulates transcription in triple-negative breast cancer and inhibits tumor growth (80, 109).

In addition, inhibition of MYC-associated transcriptional cofactors leads to reduced *MYC* mRNA expression. The bromodomain protein 4 (BRD4), a member of the bromodomain and extraterminal domain (BET) family, is an *MYC* promoter-specific coactivator containing a chromatin acetyl-lysine recognition domain that recruits transcription factor complexes such as the elongation factor p-TEFb to specific chromatin sites (15). BRD4 represents an epigenetic regulator of transcription with intrinsic kinase and HAT functions. Interference with these processes using bromodomain inhibitors leads to the inhibition of *MYC* transcription and consequently to genome-wide downregulation of MYC targets (15) (**Figure 3**), which was first demonstrated with the specific compound JQ1 (76). JQ1 releases BRD4 from chromatin and reduces *MYC* transcription and tumor growth in endometrial and ovarian cancers (77). Therefore, JQ1 or other BET inhibitors represent promising compounds to treat MYC-dependent cancers (78). This way of indirectly inhibiting MYC may offer a higher therapeutic value than direct dimerization inhibition using compounds from the first generation, which often do not display favorable pharmacokinetics and pharmacodynamics (130).

The efficacy of bromodomain inhibitors has been further improved by combining DNA methyltransferase and histone deacetylase inhibitors leading to *MYC* inactivation. This is accompanied by a reversion of immune evasion, thereby representing an innovative form of epigenetic cancer treatment (35, 131). Another type of combinatorial BRD4 inhibitor is the compound morpholinothienopyrane. In addition to inhibiting the acetyllysine binding of BRD4, this small molecule also inhibits the kinase activity of PI3K, thereby impairing PI3K/BRD4 signaling and leading to drastic downregulation of *MYC* gene expression (79) (**Figure 3**). This dual inhibition enhances MYC protein degradation leading to the inhibition of cancer cell growth and metastasis, which renders this approach promising for the development of advanced cancer therapeutics (79). In this context, a novel principle of specific protein inactivation has been established by using small hetero-bifunctional compounds termed degronimids, linking the protein of interest to a ubiquitin E3 ligase to induce proteolytic degradation (132), which can be applied for inhibiting *MYC* transcription. dBET1 represents a proteolysis-targeting chimera (PROTAC) targeted against BET family members (73), and targeted BRD4 degradation in acute myeloid leukemia (AML) by dBET1 specifically impairs *MYC* transcription. This technology has been also applied to inhibit BRD4 in Burkitt’s lymphoma cell lines (133), leading to *MYC* depletion accompanied by tumor regression in a xenograft model (74).

#### 1.4.3 Interference with MYC translation

To interfere with MYC protein biosynthesis, either destabilization of the messenger RNA (mRNA) template or targeting of critical proteins involved in *MYC* mRNA translation is aimed (109). Like with many other proteins, the translation of *MYC* mRNA is regulated by the mTORC1 complex containing the mTOR protein kinase as a catalytic subunit and by downstream substrates like the S6K1 kinase or the translation initiation factor binding protein 4E-BP1 (**Figure 3**). Unphosphorylated 4E-BP1 binds to the protein translation factor eIF4E, thereby preventing its interaction with the translation factor eIF4G. Phosphorylation of 4E-BP1 liberates a binding pocket of eIF4E and enables interaction with eIF4G and with eIF4A plus the mRNA 5′-CAP structure to initiate protein translation (109) (**Figure 3**). Consequently, pharmacological inhibition of mTORC1 with rapamycin analogs may be useful to interfere with *MYC* mRNA translation as shown previously by a reduced amount of polysome-associated *MYC* mRNA in granulocytes upon rapamycin treatment, resulting in terminal differentiation (134). The targeting of factors operating downstream of mTORC1 like the helicase eIF4A has been achieved by using silvestrol, a natural product of the flavagline family and secondary ingredient of several tropical plants (*Aglaia foveolata*). Silvestrol inhibits eIF4E, which normally unwinds mRNA secondary structures, allowing the docking of small ribosomal subunits. Therefore, MYC protein but not *MYC* mRNA levels are downregulated (109). In colon cancer where MYC is a potent oncogenic driver, inhibition of eIF4A by silvestrol reduces *MYC* translation and inhibits tumor growth in a mouse model of colorectal cancer (88). Another natural plant compound is omacetaxine (*Cephalotaxus fortunei*), an alkaloid that is used as a Food and Drug Administration (FDA)-approved drug (omacetaxine mepesuccinate) to treat tyrosine kinase inhibitor-resistant CML types. Omacetaxine inhibits protein biosynthesis by binding to the ribosomal acceptor site, thereby affecting the stability of short-lived proteins like BCR-ABL or MYC leading to cell death (86).

Since most tumor cells rely on continuous *MYC* expression, it is plausible that mRNA destabilization by short interfering RNAs (siRNAs) causes inhibition of cell proliferation and tumor regression (**Figure 3**). Appropriate *MYC* silencing using interfering RNAs may therefore represent a broadly applicable option. However, to develop clinically viable siRNA formulations for application in patients, appropriate carrier systems have to be established (135). These carrier systems can be also used for other RNA-based tools such as small guide RNAs or short hairpin RNA (shRNA) expression plasmids and are discussed below.

#### 1.4.4 Targeting MYC protein stability

In normal cells, the MYC protein is expressed at low levels and featured a short half-life, a status in which phosphorylation of the threonine 58 residue in MBI plays a critical role. This residue is mutated in the viral MYC oncoprotein (v-Myc), rendering v-Myc resistant toward GSK3β-catalyzed phosphorylation and subsequent polyubiquitination mediated by the E3 ligase FBW7 (**Figure 3**). The enzyme is a component of the SKP1/cullin-1/FBW7 E3 ubiquitin ligase complex (SCFFbw7) where the F-Box protein FBW7 mediates phosphorylation-dependent MYC degradation. However, the F-box protein SKP2 leads to MYC ubiquitination by binding to MBII, allowing differential regulation of MYC stability by targeting both MYC boxes (44). Ubiquitinated MYC is then degraded in the 26S–proteasomal complex (2, 36, 136). In addition, MYC is also phosphorylated on serine 62 by mitogen-activated protein kinases or cyclin-dependent kinases. Although this phosphorylation stabilizes the MYC protein, the modification is also needed for subsequent threonine 58 phosphorylation followed by protein phosphatase 2A (PP2A)-catalyzed dephosphorylation of serine 62. Dephosphorylation by the tumor-suppressive PP2A then finally targets MYC for proteasomal degradation (35). In addition, MYC is marked for degradation by other ubiquitin ligases such as MAGI3 which is downregulated in poor prognosis colorectal cancer. Overexpression of MAGI3 in colorectal cancer cells inhibits cell growth, promotes apoptosis, and enhances chemosensitivity to fluoropyrimidine-based chemotherapy (96). Therefore, it is possible for the activation of MYC-degrading ubiquitin ligases to interfere with MYC stability, in particular, because the MYC oncoprotein is expressed above physiological levels in MYC-dependent tumor cells. Accelerating MYC degradation by appropriate drugs and thereby significantly decreasing MYC protein levels ideally induce MYC attenuation down to normal levels to retain the normal physiological functions of MYC.

Additional proteins, which are targeted by drugs to enhance MYC degradation, have been reported. The relevant drug arsenal includes inhibitors for ubiquitin-specific protease 2B, S-phase kinase-associated protein 2, and Polo-like kinase 1 (109). Interference with functions of the proteins SET or CIP2A, which inhibit MYC-degrading PP2A, leads to increased MYC degradation (109). Another possibility for targeted MYC degradation is the application of a transcription factor targeting chimera (TRAFTAC). This molecule consists of a heterobifunctional oligonucleotide containing binding sites for the transcription factor of interest and a HaloTag-fused dCas9 protein that induces degradation of the transcription factor *via* the proteasomal pathway in a proximity-dependent manner (105). The chimeric oligonucleotide consists of a dsDNA/CRISPR-RNA chimera that recruits the E3 ligase complex through the dCas9 fusion protein *via* the RNA moiety, whereas the dsDNA portion binds MYC *via* an E-box (105). Another type of bifunctional molecule is PROTACs (see above), which are applied as anti-tumor drugs to specifically induce MYC protein degradation. These molecules could lead to efficient MYC degradation supposed that a ligand with high-affinity MYC binding is available (109). An additional example of how MYC protein stability can be impaired is given by the RAS effector protein RASSF7 having no enzymatic function but is implicated in the modulation of protein–protein interactions to regulate cell proliferation. Interestingly, RASSF7 inhibits oncogenic MYC by promoting polyubiquitination, catalyzed by the E3 ubiquitin ligase cullin 4B, which leads to MYC destabilization. Furthermore, RASSF7 competes with MAX in the formation of a heterodimer complex with subsequent attenuation of MYC target gene expression (72).

To reduce aberrant high MYC protein levels, covalent binding to MYC-interacting proteins may represent an additional option. It has been claimed that curcumin representing the principal curcumoid of turmeric (*Curcuma longa*) has anticancer properties, although its specificity, efficacy, and underlying molecular mechanisms have been controversially discussed (137, 138). Curcumin interferes with MYC-dependent cell transformation and transcriptional activation, whereby the endogenous MYC protein becomes covalently and specifically cross-linked to one of its transcriptional interaction partners, namely, the transformation/transcription domain associated protein (TRRAP), which binds to MBII in the N-terminal transactivation domain (93) (**Figure 1**). TRRAP is a component of a large complex with HAT activity and normally becomes recruited upon DNA binding of the MYC/MAX complex. Acetylation of histones then facilitates genomic accessibility and transcriptional activation (109). However, cross-linking of the transient MYC/TRRAP interaction by curcumin leads to a reduction of endogenous MYC protein levels and the cells stop to proliferate (93). Therefore, this natural spice or derivatives with higher bioavailability may constitute useful adjuvants in the therapy of MYC-dependent human tumors.

#### 1.4.5 Interference with MYC signaling to induce synthetic lethality

Proteins transmitting aberrant stimulatory signals and being involved in post-translational MYC regulation are important targets to reduce MYC activity (35). For instance, KRAS signaling mediated by the phosphatidylinositol 3-kinase (PI3K) effector pathway leads to activation of the AKT kinase and subsequent inactivation of GSK3, thereby stabilizing MYC (79). Consequently, AKT signaling inhibition leads to MYC protein destabilization as shown with the drug fludarabine phosphate, which was identified in a drug-repurposing screen on neuroendocrine prostate cancer cells (94). These cells are derived from a highly aggressive prostate cancer form featured by MYCN overexpression and loss of the tumor suppressors TP53 and RB1. Fludarabine phosphate was identified to inhibit cell proliferation by inducing reactive oxygen species (ROS) and by inhibiting AKT signaling, thereby affecting NMYC protein stability and the expression of NMYC target genes (94).

Approaches to interfere with MYC-associated upstream signaling pathways also include inhibition of kinases or phosphatases modifying critical residues in the MYC transactivation domain. Also possible is inhibiting the MYC-interacting peptidyl-prolyl isomerase PIN1, which has an impact on ubiquitin-dependent MYC proteolysis (25, 35, 65). PIN1 catalyzes proline 63 isomerization upon phosphorylation of the adjacent serine 62. The latter occurs after stimulation by activated RAS/MEK/ERK signaling or by cyclin-dependent kinases, thereby increasing MYC DNA binding and target gene regulation. Targeting protein kinases that are associated with MYC oncogenicity benefits from the fact that many clinically approved compounds are available. In general, pharmacological inhibition of critical protein kinases acting upstream of MYC could lead to reduced MYC expression by depleting oncogenic survival signals, as shown previously by simultaneous activation of PP2A and inhibition of mTOR in pancreatic adenocarcinoma (92), also demonstrating that the PI3K/AKT/mTOR pathway inhibition exhibits therapeutic activity in distinct MYC-driven cancers (22). In this context, the principle of synthetic lethality has been applied for MYC inhibition, in which a combination of two genetic events (mutations) leads to cell death, whereas a single event in one of the two genes is buffered by the other unmutated gene (109). Hence, instead of directly targeting MYC, critical proteins on which MYC depends are targeted. These proteins are usually better druggable, and normal cells with low MYC levels may remain mostly unaffected. For instance, a clinical mTOR inhibitor, blocking mTOR-dependent 4E-BP1 phosphorylation in human lymphomas, confers such synthetic lethality with MYC, demonstrating that MYC can become better druggable by applying this principle (33, 92).

In fact, oncogenic MYC offers multiple molecular and metabolic dependencies, which could be exploited to target relevant synthetic-lethal interactions (114). An example is a recent screen of more than 800 protein kinase inhibitors, which influence the stability of the MYC protein. This led to the identification of the compound UNC10112785 inhibiting CDK9, a protein kinase enhancing MYC protein stability by phosphorylating serine 62 in MBI (**Figure 3**), whereby substantial MYC destabilization was observed in pancreatic cancer cells (106). MYC is also regulated by the serine/threonine kinase PIM1 implicated in breast cancer development and progression, whose upregulation correlates with decreased patient survival and therapy resistance (139). PIM1 cooperates with MYC, leading to pronounced aggressive phenotypes. Silencing or pharmacological inhibition of PIM1 results in MYC-related tumor inactivation, suggesting an essential role of PIM1 for MYC-driven cancer (139, 140). Interestingly, *PIM3* gene encoding a PIM1 paralogue is a direct transcriptional target of MYC, suggesting a positive autoregulatory loop between MYC and PIM kinases. Likewise, PIM3 kinase potentiates the oncogenic effect of MYC leading to the acceleration of tumorigenesis, which can be blocked by the pan-PIM kinase inhibitor PIMI (25, 101).

Another enzyme directly associated with oncogenic MYC activity is RNA polymerase I catalyzing the transcription of ribosomal RNA (rRNA). High amounts of rRNA are required for the increased translational activity to support the growth and self-renewal programs of malignant cells. Selective targeting of ribosomal biogenesis by the small molecule inhibitor CX-5461 in MYC-dependent myeloma led to the inhibition of cell growth and reduction of MYC downstream target gene expression, thereby also overcoming drug resistance (141). Interestingly, in addition to decreased MYC protein translation, this compound also suppresses *MYC* mRNA levels. The latter is caused by increased binding of distinct RNA-induced silencing complexes (RISC) and the ribosomal protein RPL5 to the *MYC* mRNA transcript thereby resulting in its degradation (141).

#### 1.4.6 Modulation of MYC target genes

MYC regulates multiple genes by activating or repressing their transcription. Some of the activated MYC targets exhibit cell-transforming activity, whereas some of the downregulated genes have tumor-suppressive properties (142). Therefore, appropriate inhibition or activation of these transformation-associated targets may contribute to interfering with the growth and viability of MYC-driven cancer cells. In addition, numerous transcriptional MYC target genes represent a complex network of proteins and non-coding RNAs including long non-coding RNAs (lncRNAs) (143) and multiple microRNAs (miRNAs). For instance, MYC directly regulates the expression of several miRNAs such as the miR-17-19 cluster, miR-34a, miR-15a/16-1, and miR-9. Furthermore, the expression and activity of MYC by itself are controlled by distinct miRNAs (144). Therefore, targeting MYC-regulated miRNAs appears to represent a suitable strategy to interfere with MYC-dependent cancers, although it may be difficult to deliver miRNA mimetics into the tumor tissue of interest without losing their efficacy due to premature degradation. The possibilities to overcome these obstacles by using appropriate pharmaceutical formulations are discussed below.

In order to modify critical MYC target genes or even *MYC* itself on the nucleotide level, CRISPR-based artificial gene regulators could be applied for targeted cancer therapy, possibly also in combination with other drugs (145). The availability of the clustered regularly interspaced short palindromic repeat (CRISPR) technology to disrupt, activate, or inactivate critical genes implicated in tumorigenesis has led to the development of several approaches to selectively interfere also with oncogenic MYC functions. In addition, the CRISPR system can be applied for specific editing by changing single or multiple bases using the base or prime editing techniques (146). Normally, the CRISPR system uses the bacterial DNA-cleaving enzyme Cas9 from *Streptococcus pyogenes*, which has helicase and nuclease activities (147, 148) binding to a single-guide RNA (sgRNA). The sgRNA consists of a sequence stretch that can form Watson–Crick base pairing with the target DNA associated with characteristic protospacer adjacent motif (PAM) having the sequence 5′-NGG-3′ and representing an invariant part of the DNA target. However, it is also possible to design a programmable DNA-binding ribonucleoprotein, which can be applied as a sequence-specific DNA-binding transcription factor to specifically target a promoter of interest. In this case, a non-DNA-cleaving (dead) Cas9 (dCas9) is used in which two key amino acids are mutated (D10A and H840A). This generates a catalytically inactive enzyme (dCas9) that is linked either to a strong transcriptional activation domain (148) or to a transcriptional repressor domain (143), depending on whether a tumor-suppressive MYC target should be activated or an oncogenic MYC target downregulated.

## 2 Tools to deliver MYC inhibitors into target cells

Apart from low-molecular-weight molecules with physicochemical properties fulfilling the Lipinski criteria (149), many MYC-inhibitory molecules do not readily pass the cellular membrane like nucleic acids, antibodies, or therapeutic peptides do. Hence, a major challenge in clinical trials is to find an appropriate formulation for drug delivery to patients once an appropriate MYC inhibitor has to be tested. Efficient *in vivo* delivery must ensure that the compounds of interest are packaged adequately to be protected from sequestration, modification, or degradation before cell entry. Furthermore, it must be guaranteed that the target cell is bound by the compound or its carrier and that it translocates through the biomembrane to release its cargo into the desired cell compartment (146).

Depending on the chemical nature, different carrier systems can be used to deliver the compound into MYC-dependent tumor cells. Nucleic acids such as interfering RNAs for specific gene suppression may be applied as siRNAs or delivered by viral or non-viral DNA vectors containing coding sequences for shRNAs. Nucleic acids can be also delivered upon package into nanoparticles, as they are applied for the packaging of small organic molecules or peptides. Peptides with a cell-penetrating function may be also embedded into appropriate nanoparticles for protection from the gastrointestinal tract upon oral uptake.

In addition to using interfering RNA for specific *MYC* knockdown, tailored gene-editing techniques may lead to an even more sustained *MYC* inhibition. For the safe and efficient delivery of gene-editing agents such as *MYC*-inhibiting CRISPR components into affected organs and tissues, a variety of techniques are available, which are discussed below. The genomic editing tool CRISPR can be principally used to treat many severe diseases, but so far, its clinical applications are challenged by limitations of adequate delivery systems such as viral vectors, lipid nanoparticles, or virus-like particles (146).

### 2.1 Viral vectors

Viruses represent natural particles to deliver nucleic acids into many cell types and have been applied for gene therapeutic applications including *in vivo* gene-editing approaches (146). Due to their principal properties to infect target cells, viruses are *per se* ideal vehicles for *in vivo* delivery, making them particularly interesting and also supplying gene-editing agents. In the case of interfering RNAs, viral vectors have been applied to deliver shRNAs because of higher stability and long-term effectiveness compared to preformed 21-nt siRNA duplexes. The vector ensures that siRNAs are produced continuously within the cell leading to a prolonged knockdown of the relevant target gene. However, the application of viral vectors bears some risks in terms of immunogenicity and pathogenicity due to undesired mutations (150).

Adenovirus is a non-enveloped DNA virus with a size of approximately 100 nm that is widely applied in gene therapy trials but not yet used for broader approaches due to its potential immunogenicity and toxicity (146). A previous study showed that the expression of a *MYC* antisense RNA from an adenovirus can induce apoptosis of gastric cancer cells (151). An adenoviral vector has been also used for the coexpression of tumor suppressor genes in pancreatic cancer cells leading to proliferation inhibition and reduced MYC phosphorylation (152). Like adenovirus, adeno-associated virus (AAV) is non-enveloped but smaller in size (approximately 25 nm) and has a 5-kbp genome. AAV is relatively safe and therefore suited for the delivery of therapeutic macromolecules into clinically relevant tissues (146). With the use of this vector system, safe gene therapy applications are possible to treat multiple diseases (153). In contrast to adenovirus, lentivirus is enveloped, being derived from HIV-1 that was made replication-incompetent by deletions in the 3′-LTR. Furthermore, essential components for virus production are distributed into multiple plasmid DNA constructs (146).

### 2.2 Cell-penetrating peptides

Therapeutic peptides for cancer treatment have high target specificity and low toxicity but have limitations concerning their stabilities (154). Distinct polypeptide sequences can pass cellular membranes once they contain a so-called protein transduction domain (PTD) or a cell-penetrating sequence. Therefore, a common strategy to transport peptides into living cells is the usage of a CPP sequence. That way, the peptide of interest becomes cell-permeable in an energy- and receptor-independent manner. CPPs generally consist of less than 30 amino acids, have a net positive charge, and can be used to deliver cargos into the cytoplasm and nucleus. They have significant pharmacological potential also because of their relatively low toxicity depending on peptide concentration, cargo molecule, and coupling strategy (155). This cellular delivery system has been successfully applied to transfer small peptides into cancer cells with elevated MYC oncoprotein levels leading to a loss of viability, growth suppression, and apoptosis (156). In addition to linear cell-penetrating peptides, circularized CPPs have been proven to transport bioactive proteins into cells. The latter modification is helpful to overcome the usual endosomal uptake, which is featured by inefficient cytoplasmic release (157). Several strategies have been developed so far to design cell-permeable biologically active peptides directed against various intracellular targets (158).

Once having passed the plasma membrane, some peptides also require nuclear entry, and for this reason, methods to overcome difficulties in delivering intracellular peptides or antibodies have been developed as well. One example is *Pseudomonas* exotoxin A, which reaches the nucleoplasm *via* the endosome-to-nucleus trafficking pathway. A non-toxic truncated form of this polypeptide can be coupled to peptides to efficiently reach the nucleus. This has been successfully performed with the MYC-inhibitor peptide H1 to inhibit *MYC* transcription at a nanomolar concentration and to induce lymphoma cell killing, suggesting an interesting novel therapeutic principle against lymphoma (159).

Depending on the amino acid sequence, some peptides of interest can have intrinsic cell-penetrating properties. The widespread application of cell-penetrating peptides has driven the development of appropriate computer programs in order to predict whether a distinct polypeptide sequence has intrinsic cell membrane permeable properties. One example is the program CPPpred, which is based on a neuronal network (160). To facilitate physicochemical predictions based on the primary peptide structure, another computer program termed BChemRF-CPPred has been developed, which classifies cell−penetrating peptides using machine learning algorithms and navigating in their chemical space (161). Therefore, an artificial neuronal network is used to distinguish CPP from non-CPP properties using structure- and sequence-based descriptors extracted from common data formats.

### 2.3 Nanoparticles

Peptides, nucleic acids, or other drug molecules that do not pass the cellular barrier require specific formulations for appropriate and selective delivery into MYC-dependent tumor cells. This is particularly important for nucleic acids because their direct delivery without chemical modifications is hampered by the fact that naked nucleic acids are rapidly digested by serum nucleases. Furthermore, size and negative charge prevent nucleic acids from being passaged through biomembranes, which makes nano-sized delivery systems attractive, where nucleic acids electrostatically associate with positively charged molecules (135). Therefore, to safely apply DNA or RNA as therapeutic agents for the treatment of various diseases, safe, effective, and stable delivery systems are required to protect nucleic acids from degradation and to ensure efficient cellular uptake (162).

**Figure 4:**
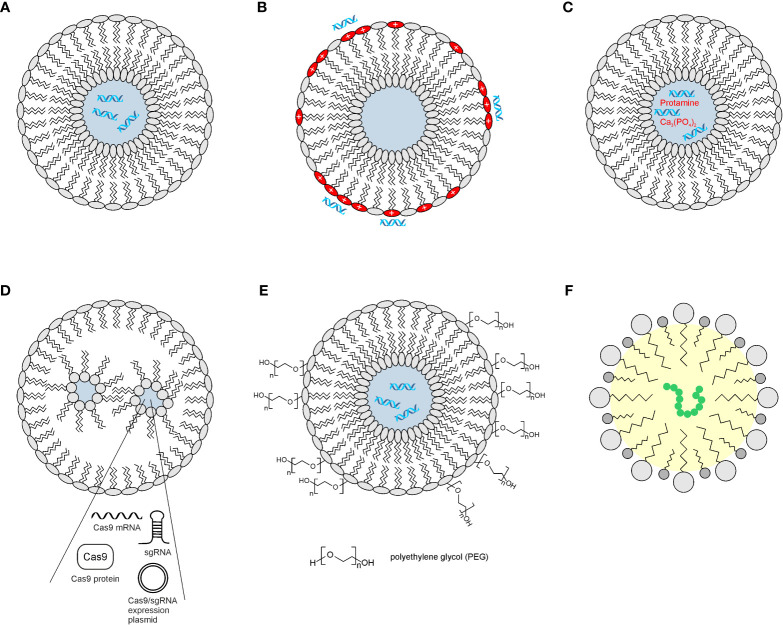
Examples of lipid-based nanocarriers. Cross-sections of spherical nanoparticles are used to encapsulate nucleic acids such as siRNAs, mRNAs, plasmids, or polypeptides. Hydrophilic and lipophilic areas are shaded in blue and yellow, respectively. **(A)** Neutral liposome carrying siRNAs. **(B)** Cationic liposome with negatively charged siRNAs at the surface. **(C)** Liposome with positively charged matrix in the interior complexed with negatively charged siRNAs. **(D)** Lipid nanoparticle encapsulating components of the CRISPR/Cas9 system, which consist either of expression plasmids, RNA, or protein. **(E)** Neutral liposome covered with hydrophilic polyethylene glycol for enhanced dispersion. **(F)** Self-nanoemulsifying drug delivery system (SNEDDS) representing an isotropic mixture of oil, surfactant (dark gray), and co-surfactant (light gray) for generation of an oil-in-water emulsion.

Nanoparticles, which are defined as objects with a diameter of between 1 and 100 nm, have been developed in recent years to transport therapeutic cargos of interest and to overcome cellular barriers. Ideally, formulations are used, which can be also applied for personalized applications, thereby improving precision therapies (163). The advantages of this delivery form are simplicity, self-assembly, biocompatibility, and bioavailability, which render them the most approved class of nanomedicinal products (163). According to their different structures, nanoparticles are lipid-based, polymeric, or inorganic. Biocompatible polymeric nanoparticles are made of natural or synthetic monomers and are generated by emulsification, nanoprecipitation, or ionic gelation. Therefore, the drug of interest is packaged within the polymeric matrix, chemically conjugated, or bound to the nanoparticle surface (163) (**Figure 4**).

Liposomes are a subset of lipid-based nanoparticles and are composed of amphipathic phospholipids, which carry and deliver both hydrophilic and hydrophobic drugs. They are rapidly taken up by the reticuloendothelial system and consist of a spherical self-assembled phospholipid bilayer with a hydrophilic aqueous interior in which water-soluble substances are entrapped (163) (**Figure 4**). Liposomes have been used to encapsulate *MYC*-specific siRNAs. A neutral liposome consisting of dioleoylphosphatidylcholine (DOPC), cholesterol (Chol), and distearoylphosphatidylethanolamine polyethylene glycol (DSPE-PEG) was applied to deliver a *MYC*-specific siRNA into ovarian xenograft tumors leading to reduced growth (164). Polyethylene glycol (PEG) generates a hydration shell around the liposome to sterically prevent interparticle associations (165). Likewise, synthetic cationic liposomes consist of a cationic lipid and a zwitterionic phospholipid, thereby creating vesicles that bear a net positive charge. The advantage of nucleic acid transport is that those liposomes electrostatically associate with negatively charged molecules on the surface, and thus, the nucleic acid does not have to become encapsulated. In the case of the widely applied Lipofectamine™, the liposomes are prepared by mixing equimolar quantities of the cationic lipid *N*,*N*-dimethylaminopropyl-amidosuccinyl-cholesterylformylhydrazide and cholesterol. Successful downregulation of *MYC* mRNA and protein has been demonstrated using Lipofectamine™-mediated delivery of siRNA in colon cancer cells, leading to proliferation inhibition, induction of apoptosis, and cell growth suppression (166). In addition, multiple liposome formulations to package *MYC*-specific siRNAs have been described such as liposome-polycation-DNA nanoparticles consisting of 1,2-dioleoyl-3-trimethyl-ammonium-propane (DOTAP) and cholesterol to envelop a core of protamine-bound nucleic acid and calf thymus DNA, or lipid calcium phosphates (135) (**Figure 4**).

Similar to liposomes, lipid nanoparticles (LNPs) represent spherical platforms consisting of a lipid bilayer, which surrounds an aqueous compartment (162) (**Figure 4**). However, LNPs differ from liposomes by forming micellar structures within the particle core consisting of four major components. One of them is cationic lipids, which are complex with negatively charged nucleic acids. Other components are phospholipids, cholesterol, and PEGylated lipids required for particle structure, stability, and membrane fusion. The efficacy of nucleic acid delivery makes LNPs particularly important for personalized genetic applications because they have a neutral charge during delivery but become charged in the endosome leading to endosomal escape (163). LNPs have been proven useful for the delivery of interfering RNAs to inhibit the growth and viability of MYC-dependent tumor cell lines or tumors. In addition, siRNAs directed against *MYC* mRNA, DICER-substrate siRNA, and shRNA expression plasmids have been applied using organic and inorganic nanoparticles (135).

Difficulties such as low transfection efficiency, low control of integration into the host DNA, and unstable expression have been overcome by the usage of the macromolecule polyglycidalmethacrylate (PGMA) as a platform to graft multiple cationic polyethyleneimine (PEI) chains, which bind negatively charged nucleic acids such as microRNAs or plasmid DNAs encoding short interfering RNAs (150). These nanoparticles were orally delivered into breast and colon cancer transgenic mouse models, leading to increased survival. Furthermore, RNA interference using these conjugated nanoparticles carrying microRNAs directed against *MYC* suppressed the transformed phenotype in relevant breast and colorectal cancer cell lines with an efficiency comparable to virally based systems (150). Other approaches for *MYC* inhibition have been carried out using nanoparticles with anisamide-targeted *MYC*-specific siRNA to enter melanoma cells upon binding to a cell-specific sigma receptor using murine and human xenograft tumor models (167). Even more effective were nanoparticles composed of a guanidinium derivate containing a cationic lipid, which led to sensitizing of the tumor cells toward paclitaxel (167). Another example is the usage of a cationic lipid nanoparticle encapsulating an anti-miR-17 oligonucleotide in a conditional transgenic mouse model of MYC-driven hepatocellular carcinoma leading to decreased cell proliferation, apoptosis, and delayed tumorigenesis (168). A further application of a shRNA expression plasmid was explored in a study where double-emulsion nanoparticles with *MYC*-specific siRNA fragments and plasmids were delivered into glioma cancer cells in order to interfere with *MYC* expression, which then led to programmed cell death (169).

Apart from interfering with RNAs, various gene-editing techniques have been employed to induce gene disruption or modification. In particular, CRISPR technology is an attractive tool to interfere with oncogenic MYC functions. However, a major challenge is to find an adequate delivery technology for clinical translation into cancer therapy. To apply the required CRISPR/Cas9 molecules in form of a Cas9 or dCas9 mRNA plus appropriate single-guide RNAs, lipid nanoparticles can be used (170) (**Figure 4**). These nanovesicles, based on lipids, polymers, peptides, or extracellular vesicles, increase Cas9 and sgRNA delivery through endosomal escape (171). Modified lipid nanoparticles have been applied for efficient delivery of CRISPR/Cas9-relevant ribonucleoprotein particles (RNPs) into cells upon intravenous injection (172), and the first clinical studies have been published (173). With the use of CRISPR-mediated activation (CRISPRa), tumor suppressor genes *SERPINB5* (*MASPIN*) and *CCN6* were reactivated by intravenous delivery of a nanoscale dendritic macromolecular delivery agent (174). Hence, this technology might be useful to reactivate potential tumor suppressor genes like *BASP1*, whose promoter is silenced by methylation in MYC-dependent acute myeloid leukemia (175).

In addition to delivery *via* synthetic lipid vesicles, it is worth mentioning that siRNA or CRISPR/Cas9 components can also be loaded into exosomes. Exosomes are membrane-bound extracellular vesicles with a lipid bilayer similar to liposomes, which are present in biological fluids of multicellular organisms. Exosomes are naturally released by cells for the purpose of intercellular communication and represent an emerging nanocarrier system for a variety of medically relevant molecules (135). In fact, the potential for exosome-mediated anti-*MYC* siRNA delivery was recently demonstrated (176). Furthermore, when modified with a chimeric antigen receptor (CAR), selective tropism is given leading to specific particle accumulation in tumor cells, followed by CRISPR/Cas9 system release and subsequent targeting of the *MYC* oncogene in lymphoma (177). In addition, there are naturally occurring nanoparticles with exosome-like structures. An example of this is the nanoparticle GaELN from the garlic plant (*Allium sativum*) (178). GaELN particles are useful to reverse high-fat-diet-induced obesity in mice in which inflammatory processes play an important role. Orally administered GaELNs are taken up by microglial cells leading to inflammation inhibition whereby the phosphatidic acid component interacts with the neuronal signaling effector brain acid-soluble protein 1, which is encoded by downregulated MYC target gene *BASP1* (17). The GaELN/BASP1 complex inhibits MYC by competitive binding to calmodulin (CaM) (178), as it has been reported previously in v-*myc* transformed fibroblasts (179). Subsequent expression inhibition of the MYC target STING then leads to reduced expression of several inflammatory cytokines including IFN-γ and TNF-α (178).

Additional nanoparticle applications to deliver small molecules for MYC inhibition have been described. In esophageal cancer, MYC is aberrantly activated due to *MYC* gene amplification. Synthetic lethal interactions between MYC signaling and small-molecule inhibition involved in cell cycling have been therapeutically addressed to selectively kill tumor cells (180). Therefore, the flavonoid alkaloid CDK inhibitor alvocidib was applied in combination with nanoparticle albumin-bound paclitaxel to interfere with cell proliferation. Another example of nanotherapeutic delivery is the transport of a MYC inhibitor into tumor-associated macrophages in breast cancer (64). In this case, a MYC inhibitor prodrug termed MI3-PD encapsulated within perfluorocarbon nanoparticles was delivered directly into the cytosol of the target cell through a phagocytosis-independent mechanism (64).

Nanoparticles can be also applied to deliver natural plant compounds like curcumin, which inhibits MYC-specific transactivation and cell transformation (93). To overcome the problem of curcumin’s low bioavailability due to its highly hydrophobic character (181), solid lipid nanoparticles used for encapsulation enhance the bioavailability and stability of this drug, thereby improving its therapeutic potential in MYC-dependent tumors (182).

Concerning nanoparticles to package MYC-inhibiting peptides, small protein scaffolds with anionic polypeptides providing electrostatic interactions with the positively charged lipid-based delivery system have been developed (183). As proof of principle, the MYC-inhibitory peptide Omomyc was fused C-terminally to a negatively charged polypeptide, giving rise to efficient membrane penetration and blocking of MYC-dependent transcription in lung cancer cells (183).

Due to poor oral bioavailabilities, most therapeutic peptides have to be administered parenterally, which is inconvenient and painful and results in lower patient compliance. Although oral peptide application is favored, this is hampered by multiple gastrointestinal barriers like the mucus covering gastrointestinal epithelial cells, peptide-degrading enzymes, or a sulfhydryl barrier mediated by high glutathione concentrations leading to disulfide exchange reactions and drug inactivation (184). To overcome these difficulties, lipid-based nanocarriers such as oil-in-water nanoemulsions, self-nanoemulsifying drug delivery systems (SNEDDS), solid lipid nanoparticles (SLNs), nanostructured lipid carriers (NLCs), liposomes, or micelles are used as carrier systems. Of particular interest are SNEDDS consisting of an isotropic mixture of oil, surfactant, and a co-surfactant (**Figure 4**). Upon mixture with water, a thermodynamically stable oil/water nano-emulsion is formed with droplet sizes of 20–200 nm, which is ideal to enclose lipophilic peptides. The lipophilic character of SNEDDS nanoparticles with sizes below 200 nm and a muco-inert surface provided by polyethylene glycol favors permeation across epithelial cells of the gastrointestinal tract. Subsequently, nanoparticles are resorbed upon cell membrane fusion or endocytosis releasing their load into the systemic circulation (185). In addition to peptides, small hydrophobic molecules like curcumin can be emulsified into such nanoparticle type, which leads to significantly increased bioavailability (186).

## 3 Outlook

To effectively inhibit aberrantly activated MYC in tumor tissue, innovative strategies and advanced carrier concepts are required. In order to precisely treat MYC-dependent cancers by oral drug administration, special measures are required to enhance drug bioavailability and patient compliance. In addition to the development of an efficient inhibitor that targets an oncogenic transcription factor such as MYC, the definition of an appropriate pharmaceutical formulation for specific delivery represents a further challenge. The spectrum of treatment options for cancer such as surgery, radiation, chemotherapy, or drugs specifically targeting oncogenic molecules has to be complemented by the usage of nanostructures, which interact with target tumor cells, thereby significantly reducing undesired side effects. Nanotechnology thus could provide the missing link between the development of novel treatment principles obtained from basic research and suitable pharmaceutical technologies for application in patients. Nanoparticles with sizes in the range of larger biomolecules can encapsulate small molecule compounds and are able to enter tumor cells, thereby benefitting from the enhanced permeabilization of many tumor cell types. In addition, nanoparticles can be chemically modified on the surface to increase stability and circulation time to reach tumor cell types, which are not easily accessible by passive transport. The active targeting of nanoparticles into MYC-dependent tumor cells or specific intracellular compartments can be achieved by appropriate surface modifications that are relevant to the development of next-generation nanoparticles. Furthermore, nanoparticles have the advantage that they are relatively safe and because their programmability enables personalized medicine, which is of utmost importance in precision oncology.

## Author contributions

MH selected the topic, and MH and LW designed the outline of the review. LW and MH wrote the article. All authors contributed to the article and approved the submitted version.

## Glossary

**Table:**

4E-BP1	eukaryotic translation initiation factor 4E-binding protein 1
AAV	adeno-associated virus
Ac	acetylation
ALL	acute lymphoblastic leukemia
AML	acute myeloid leukemia
BASP1	brain acid-soluble protein 1
bCTNN	β-catenin
BET	bromodomain and extraterminal domain
bHLH-LZ	basic helix-loop-helix-leucine zipper
BRD4	bromodomain protein 4
CaM	calmodulin
CAMKIIγ	Ca^2+^/calmodulin-dependent protein kinase II γ
CAR	chimeric-antigen receptor
CCN6	cellular communication network factor 6
CDK	cyclin-dependent kinase
CDT1	chromatin licensing and DNA replication factor 1
CIP2A	cancerous inhibitor of PP2A
CML	chronic myeloid leukemia
CPP	cell-penetrating peptide
CRISPR	clustered regularly interspaced short palindromic repeat
CRPC	castration-resistant prostate cancer
C-terminus	carboxl-terminus
DOTAP	1,2-dioleoyl-3-trimethyl-ammonium-propane
DSPE-PEG	distearoylphosphatidylethanolamine polyethylene glycol
E-box	enhancer box
ECM	extracellular matrix
EGF	epidermal growth factor
eIF4	eukaryotic translation initiation factor 4
EMSA	electrophoretic mobility shift assays
ER	estrogen receptor
ERK	extracellular-signal regulated kinase
FBP	FUSE-binding proteins
FDA	US Food and Drug Administration
FIR	FBP-interacting repressor
FPPa	penetrating phylomer peptide
FZD	frizzled
GRB2	growth factor receptor-bound protein 2
GSK3	glycogen synthase kinase 3
HAT	histone acetyltransferase
HDAC	histone deacetylase
IFN-γ	interferon γ
IGF	insulin-like growth factor
IRS	insulin receptor substrate
JAK	Janus kinase
lncRNA	long non-coding RNA
LNP	lipid nanoparticle
LTR	long terminal repeat
MAPK	mitogen-activated protein kinase
MAX	MYC-associated factor X
MB	MYC
MEK	MAPK/ERK-Kinase
miRNA	microRNA
MIZ-1	MYC-interacting zinc finger 1
mTOR	mammalian target of rapamycin
MYC	myelocytomatosis oncogene
NLC	nanostructured lipid carrier
N-terminus	amino-terminus
ORI	origin of replication
p-TEF	positive transcription elongation factor
PAM	proto-spacer adjacent motifs
PDB	Protein Data Bank
PDK	phosphoinositide-dependent protein kinase
PEG	polyethylene glycol
PEI	polyethyleneimine
PGMA	polyglycidalmethacrylate
PI3K	phosphatidylinositol-3-phosphate kinase
PIN1	peptidyl-prolyl *cis*–*trans* isomerase NIMA-interacting 1
PIP2	phosphatidylinositol 4,5-bisphosphate
PIP3	phosphatidylinositol (345)-trisphosphate
PNA	peptide nucleic acid
PP2A	protein phosphatase 2A
PPI	protein–protein interactions
PR	progesterone receptor
PROTAC	proteolysis-targeting chimera
PTD	protein transduction domain
RB	retinoblastoma protein
RISC	RNA-induced silencing complex
RNP	ribonucleoprotein particle
ROS	reactive oxygen species
RTK	receptor tyrosine kinase
S6K	S6 kinase
sgRNA	single-guide RNA
shRNA	short hairpin RNA
siRNA	short interfering RNA
SLN	solid lipid nanoparticle
SNEDDS	self-nanoemulsifying drug delivery systems
STAT	signal transducer and activator of transcription
TBP	TATA box binding protein
TF	transcription factor
TGFbR	transforming growth factor β
TNBC	triple-negative breast cancer
TNF-α	tumor necrosis factor α
TRAFTAC	transcription factor targeting chimera
TRRAP	transformation/transcription domain-associated protein
TSC	tuberous sclerosis complex
WT1	Wilms tumor suppressor protein 1
